# An iron chelation-based combinatorial anticancer therapy comprising deferoxamine and a lactate excretion inhibitor inhibits the proliferation of cancer cells

**DOI:** 10.1186/s40170-022-00284-x

**Published:** 2022-05-12

**Authors:** Koichi Fujisawa, Taro Takami, Toshihiko Matsumoto, Naoki Yamamoto, Takahiro Yamasaki, Isao Sakaida

**Affiliations:** 1 Department of Gastroenterology and Hepatology, Yamaguchi University Graduate School of Medicine, Minami Kogushi 1-1-1, Ube, Yamaguchi, 755-8505 Japan; 2 Department of Environmental Oncology, Institute of Industrial Ecological Sciences, University of Occupational and Environmental Health, Kitakyushu, Fukuoka, 807-8555 Japan; 3 Department of Oncology and Laboratory Medicine, Graduate School of Medicine, Yamaguchi University, Minami Kogushi 1-1-1, Ube, Yamaguchi, Japan; 4 Yamaguchi University Health Administration Center, Yamaguchi, Japan

**Keywords:** Hypoxia, Antitumor effect, Iron chelator, Energy metabolisms, Lactate, Glutaminase, Autophagy

## Abstract

**Background:**

Although iron chelation has garnered attention as a novel therapeutic strategy for cancer, higher levels of efficacy need to be achieved. In the present study, we examined the combinatorial effect of deferoxamine (DFO), an iron chelator, and α-cyano-4-hydroxy cinnamate (CHC), a suppressor of lactate excretion, on the proliferation of cancer cell lines.

**Methods:**

We established a deferoxamine (DFO)-resistant cell line by culturing HeLa cells in media containing increasing concentrations of DFO. Metabolome and gene expression analyses were performed on these cells. Synergistic effect of the drugs on the cells was determined using an in vitro proliferation assay, and the combination index was estimated.

**Results:**

DFO-resistant HeLa cells exhibited enhanced glycolysis, salvage cycle, and de novo nucleic acid synthesis and reduced mitochondrial metabolism. As DFO triggered a metabolic shift toward glycolysis and increased lactate production in cells, we treated the cancer cell lines with a combination of CHC and DFO. A synergistic effect of DFO and CHC was observed in HeLa cells; however, the same was not observed in the human liver cancer cell line Huh7. We hypothesized that the efficacy of the combination therapy in cancer cells depends on the degree of increase in lactate concentration upon DFO treatment.

**Conclusion:**

Combination therapy involving administration of DFO and CHC is effective in cancer cells wherein DFO treatment results in an elevation in lactate levels. Our findings illustrate that the DFO-induced enhanced glycolysis provides specific targets for developing an efficient anticancer combinatorial therapy involving DFO. These findings will be beneficial for the development of novel cancer chemotherapeutics.

**Supplementary Information:**

The online version contains supplementary material available at 10.1186/s40170-022-00284-x.

## Introduction

Iron is an essential trace element for the body, and several iron-containing proteins, such as HIF-hydroxylase, a member of the 2-oxoglutarate dioxygenase family, and collagen hydroxylase, and other enzymes involved in carnitine synthesis are garnering attention [1].

The mechanism of iron metabolism and iron-derived oxidative stress in cancer has been analyzed in detail in recent years, highlighting the value of regulating iron metabolism [2, 3]. High concentrations of iron are required by rapidly growing neoplastic cells; therefore, iron removal has been considered a novel therapeutic strategy for cancer. The most well-known iron chelator, deferoxamine mesylate (DFO), is derived from *Streptomyces pilus*; DFO is a hexadentate siderophore with high affinity to iron [4]. Administration of DFO and the subsequent chelation of iron inside cells result in the inhibition of iron-dependent enzyme activities. Metabolic changes of particular interest occurring in iron-deprived cells include mimicking of a hypoxia-like condition via HIF1α accumulation. This occurs as a result of reduced activity of the prolyl hydroxylase domain-containing (PHD) protein, which is responsible for the hydrolysis of HIF1α, and growth inhibition mediated by the reduced activity of the iron-dependent ribonucleotide reductase [5]. We have previously demonstrated DFO-mediated suppression of the growth of preneoplastic lesions in rat liver [6]. Additionally, the clinical efficacy of iron chelators has been demonstrated in pilot studies involving advanced hepatocellular carcinoma patients [7, 8], and the efficacy of the orally administered iron chelator deferasirox (DFX) for pancreatic cancer has also been reported [9].

Thus far, it has been reported that a combinatorial anticancer drug therapy using an iron chelator, specifically DFO, and arsenic trioxide, shows a synergistic effect in leukemia cells, without any adverse effects [10]. It has also been shown in several cell lines that the combination of DFX with doxorubicin, cisplatin, or carboplatin inhibits cell growth and induces apoptosis [11, 12]. We have reported that a combination of DFX and gemcitabine is effective against pancreatic cancer cell lines [13]. However, previous studies have primarily investigated the combinations of DFO with frequently used anticancer drugs, and there have been no reports on the metabolic changes caused by DFO in different cancer cells.

As many iron-containing proteins may serve as targets of iron chelation, it is difficult to evaluate in detail the precise effect of DFO. Additionally, it is necessary to know which targets are important in each cell type. Therefore, in this study, we generated a DFO-resistant strain of HeLa cells that has not been reported thus far and analyzed the metabolic changes occurring in these cells in the presence and absence of DFO. We also aimed to evaluate the changes caused by DFO administration in nonresistant strains and identify which therapeutic target is important for the survival of cancer cells.

## Methods

### Cell culture

HeLa cells (JCRB9004) and Huh7 cells (JCRB0403) were obtained from the Japanese Collection of Research Bioresources (Osaka, Japan) and cultured in Dulbecco’s Modified Eagle’s Medium (DMEM) (Gibco, Japan) supplemented with 10% fetal bovine serum (SAFC, MO, USA). The DFO-resistant cell lines were established by gradually increasing the DFO concentration (started from 3 μM) over a duration of approximately 6 months. Cells (2000~6000/well) were seeded in 96-well plates and treated with different concentrations (0~100 μM) of DFO, and cell proliferation was determined by measuring the area of the cells using the Incucyte HD imaging system (Essen BioScience, Ann Arbor, MI). The combination activity of the drugs was estimated with the CalcuSyn software program (Biosoft, Ferguson, MO). Briefly, this program determines the combination index (CI), a quantitative measure of the degree of drug interactions. The information related to drugs is provided in Supplemental Table 1.

### Western blotting

Western blotting was performed using standard methods [14]. Briefly, cells were lysed in lysis buffer (62.5 mM Tris-HCl (pH 6.8), 4% sodium dodecyl sulfate (SDS), and 200 mM dithiothreitol). SDS-PAGE was performed on a 12% acrylamide gel by loading 20 μg of sample protein per well; this was followed by electrotransfer of the proteins onto polyvinylidene fluoride (PVDF) membranes (BioRad, Tokyo, Japan) and blocking of nonspecific epitope binding using 5% skim milk. Then, membranes were probed using primary antibodies (1 h) at room temperature. Information on primary antibodies is provided in Supplementary Table 1.

### Lactate measurement

Lactate in cell culture supernatant (24 h after DFO administration) was measured using a lactate pro2 sensor (Arkley, Kyoto, Japan) and corrected using CyQUANT (Life Technologies). Intracellular lactate concentration (24 h after DFO administration) was measured from cell extracts and corrected using protein concentration.

### Metabolome analysis

Metabolome and statistical analyses were conducted at Metabolon as described previously [15]. Briefly, cell pellets were subjected to methanol extraction; the extract was then divided into aliquots for analysis by ultrahigh performance liquid chromatography/mass spectrometry (UHPLC/MS) in the positive, negative, or polar ion mode and by gas chromatography/mass spectrometry (GC/MS). Metabolites were identified by comparing ion features to a reference library of chemical standards using an automated method, followed by visual inspection for quality control. For statistical analyses and data display, any missing values were assumed to be below the limits of detection; these values were imputed with the compound minimum.

### Total RNA isolation

Total RNA was isolated from cerebellums of each individual sample using TRIzol Reagent (Life Technologies) and purified using SV Total RNA Isolation System (Promega) according to the manufacturer’s instructions. RNA samples were quantified on an ND-1000 spectrophotometer (NanoDrop Technologies, Wilmington, DE), and RNA quality was confirmed using the Experion System (Bio-Rad Laboratories, Hercules, CA).

### Serial analysis of gene expression (SAGE)

Ion AmpliSeq Transcriptome Human Gene Expression Kit (Life Technologies) was used for library creation [16]. An Ion Proton next-generation sequencer library of analysis beads was created, and an Ion PI IC 200 Kit (Life Technologies) and Ion PI Chip Kit v2 BC were used for sequencing on an Ion Proton next-generation sequencer. The results of metabolome analysis and SAGE were integrated by ingenuity pathways analysis (IPA).

### Oxygen consumption rate (OCR) measurements

OCR measurements were performed using a Seahorse Biosciences XF96 Extracellular Flux Analyzer. Cells were seeded at a density of 10,000 cells/well in XF96 microplates (Seahorse Biosciences). After a 24-h incubation, the growth medium was replaced by XF assay medium (Seahorse Biosciences) supplemented with 25 mM glucose (Sigma-Aldrich). OCR measurements were made over 5-min periods following a 3-min mix period. Cells were treated by sequential addition of 1 μg/mL oligomycin (Sigma-Aldrich), 300 nM carbonylcyanide-*p*-trifluoromethoxyphenylhydrazone (FCCP; Sigma-Aldrich), and 2 μM rotenone (MP Biomedicals). The spare respiratory capacity and coupling efficiency were calculated in accordance with the Seahorse Bioscience instructions, and the basal OCR was normalized to the cell number.

### Statistical analysis

The results were analyzed using either the two-tailed unpaired Student’s *t*-test or Welsh’s two-factor *t*-tests; data are presented as mean ± standard deviation, with significance level established at *p* < 0.05. One-way ANOVA, followed by the Tukey–Kramer or Steel-Dwass test, was used for comparison of more than two groups. The significance of metabolome analysis was determined with Array Studio (OmicSoft) or “R” to compare protein-normalized data between experimental groups; *p* < 0.05 was considered significant. ANOVA contrasts were used to identify biochemicals that differed significantly between experimental groups.

## Results

### Treatment of HeLa cells with DFO reduces growth and mitochondrial activity

**Fig. 1:**
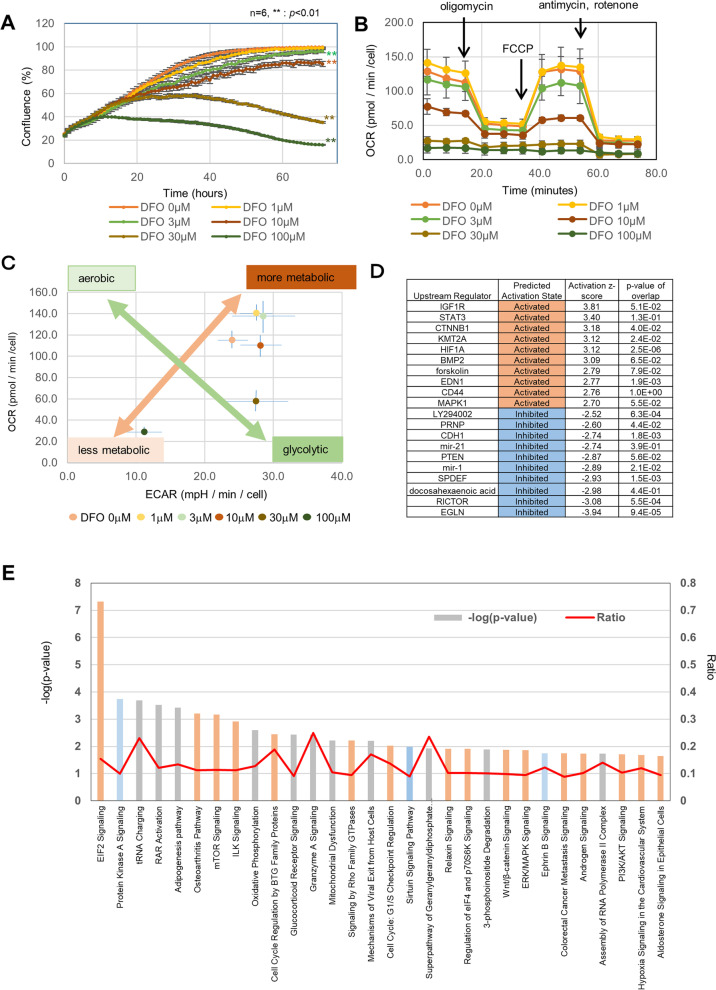
Evaluation of the impact of DFO on HeLa cells. **A** Measurement of the effect of DFO on cell proliferation. **Indicates *p* < 0.01 compared to control group (one-way ANOVA followed by Tukey’s post hoc test). Cell proliferation was determined by measuring the area of the cells using the Incucyte HD imaging system. **B** Evaluation of OCR using a flux analyzer. 1 μM oligomycin, 0.5 μM FCCP, 1 μM antimycin, and 1 μM rotenone were added to the wells. **C** Metabolic phenogram. Basal OCR and ECAR rates were plotted in response to a 48-h DFO treatment in HeLa cells. The top-left corner of the figure indicates aerobic, bottom-right corner indicates glycolytic, top-right corner shows activated metabolism, and bottom-left corner shows reduced metabolism. Values represent mean ± SD. **D** A list of top five and bottom five upstream regulators. Presumed activated regulators are shown in brown, whereas those presumed inhibited are shown in blue. Cells were treated with 30 μM of DFO for 2 days. **E** Pathway ranking by IPA analysis. Reciprocal display of *p*-value calculated using the IPA software; magenta line is the ratio of genes included in each pathway. Cells were treated with 30 μM of DFO for 2 days

We evaluated the impact of DFO on cell growth using HeLa cells, which are widely used in cancer research. DFO suppressed cell growth in a concentration-dependent manner. In particular, remarkable growth suppression was observed at 100 μM or higher concentrations of DFO (Fig. 1A). Evaluation of oxygen consumption rate (OCR), using a flux analyzer, revealed a decrease in OCR with increased DFO concentration (Fig. 1B). In addition, evaluation of the extracellular acidification rate (ECAR)/OCR ratio indicated a tendency towards glycolytic metabolism and overall reduced metabolism with increasing DFO concentrations (Fig. 1C). The changes in gene expression in response to DFO treatment were evaluated with SAGE and IPA. The results indicated that iron chelation was an upstream event that led to the accumulation and activation of HIF1α and downregulation of EGLN (Egl nine homolog) (Fig. 1D). Pathway analysis identified changes in EIF2 signaling, tRNA charging, suggesting decreased protein synthesis, and in processes related to cell proliferation, such as cell cycle checkpoint regulation, mitochondrial dysfunction, and oxidative phosphorylation, in addition to hypoxia signaling (Fig. 1E).

### Enhanced salvage cycle, de novo nucleic acid synthesis, and glycolysis and reduced mitochondrial metabolism are beneficial for HeLa cell survival under DFO treatment

**Fig. 2:**
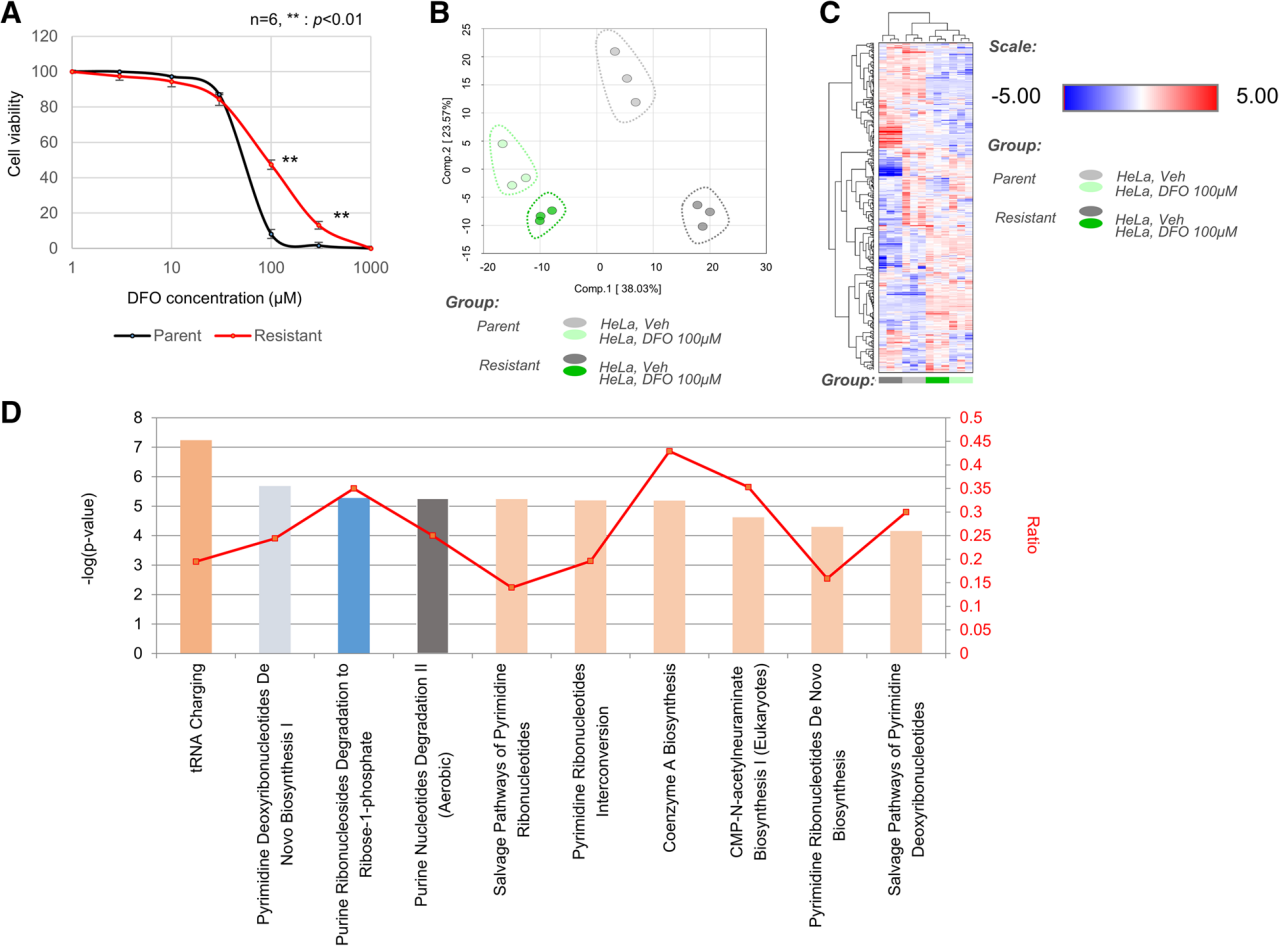
Analysis of DFO-resistant HeLa cells. **A** Evaluation of the degree of resistance of DFO-resistant cell lines. The statistical differences are indicated by **< 0.01 (Student’s *t*-test). **B** Principal component analysis-normalized metabolic data. Percentage values indicated on the axes represent the contribution rate of the first (PC1) and second (PC2) principal components to the total amount of variation. Cells were collected on 2 days after DFO treatment and analyzed. **C** Heat map of the hierarchical cluster analysis. The rows show the normalized levels of each metabolite. The dendrogram for each heatmap shows the relatedness of the normalized metabolite level patterns. **D** Pathway ranking by IPA. Reciprocal display of *p*-value calculated using the IPA software; magenta line is the ratio of metabolites included in each pathway

**Fig. 3:**
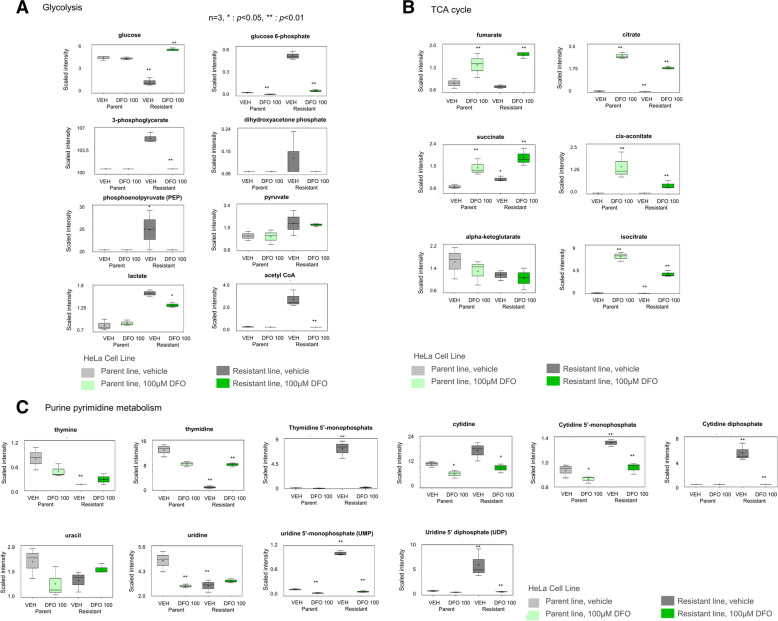
Changes in metabolites related to glycolysis, TCA cycle, and purine/pyrimidine metabolism of DFO-resistant HeLa cells. **A** Changes in glycolytic metabolites according to metabolome analysis (shown on the right). Schematic representation of the glycolytic pathway on the left. Cells were collected 2 days after DFO treatment and analyzed. The statistical differences between parent VEH vs parent DFO100, parent VEH vs resistant VEH, and resistant VEH vs resistant DFO100 are shown by *< 0.05 and **< 0.01 (ANOVA contrasts). **B** Changes in TCA cycle metabolites according to metabolome analysis. **C** Changes in purine and pyrimidine metabolism according to metabolome analysis

As various types of enzymes require iron for their activity, it is difficult to evaluate in detail the effect of DFO on cells. We generated a DFO-resistant HeLa strain and analyzed the underlying mechanisms of DFO resistance. The DFO-resistant HeLa cell line was established by gradually increasing the DFO concentration (starting from 3 μM) over a duration of approximately 6 months (Fig. 2A). Metabolome analysis was performed to evaluate metabolic changes in the parent strain and the DFO-resistant strain with and without treatment with 100 μM DFO. PCA analysis clearly distinguished four groups, comprising the parent DFO non-treated group (parent Veh), parent 100 μM DFO-treated group (parent DFO), resistant DFO non-treated group (resistant Veh), and resistant 100 μM DFO-treated group (resistant DFO) (Fig. 2B). Furthermore, hierarchical clustering also identified clear variations among the four groups (Fig. 2C). In addition, we performed IPA of the gene expression data from SAGE analysis, in combination with that from metabolome analysis, to compare the parent Veh and resistant Veh groups. Pathways related to purine and pyrimidine salvage cycles, de novo synthesis, and tRNA charging were identified as the top canonical pathways (Fig. 2D). Metabolome analysis to compare the metabolites related to glycolysis among parent Veh and resistant Veh groups showed that the levels of glucose-6-phosphate, 3-phosphoglycerol, acetyl-CoA, pyruvate, and lactate increased in the resistant Veh group (Fig. 3A). Furthermore, both parent and resistant strains showed accumulation of TCA cycle metabolites upon DFO treatment. Comparison of the parent DFO and resistant DFO groups revealed significant decrease in the levels of citrate, cis-aconitate, and isocitrate in the resistant DFO group (Fig. 3B). Additionally, with respect to purine- and pyrimidine-related metabolites, the resistant DFO group showed increased TMP, UMP, and UDP levels and decreased thymidine, thymine, and uridine levels (Fig. 3C).

### DFO and lactate excretion inhibitor exhibit a synergistic effect in HeLa cells

**Fig. 4:**
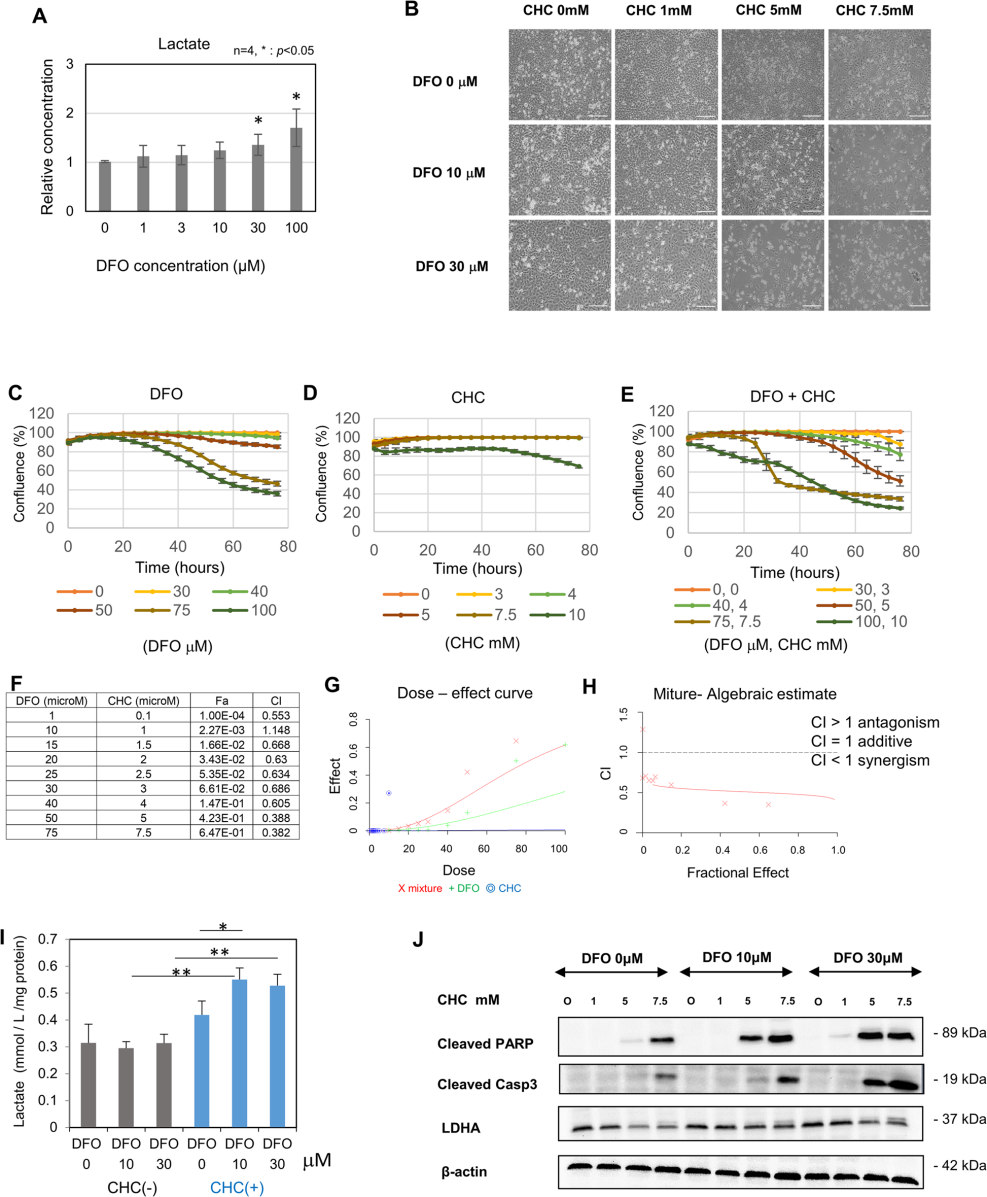
Evaluation of the effect of combined DFO + CHC treatment in HeLa cells. **A** Evaluation of secreted lactate accumulation in the culture medium according to changes in DFO concentration (day 2). *Indicates *p* < 0.05 compared to control group (one-way ANOVA followed by Tukey’s post hoc test). **B** Changes observed in cell culture after DFO + CHC treatment — 72 h after administration. Scale bar, 300 μm. **C** Changes in cell confluence after DFO monotherapy. **D** Changes in cell confluence after CHC monotherapy. **E** Changes in cell confluence after DFO + CHC treatment. **F** Combination index of the DFO + CHC treatment. Fa, fraction affected; CI, combination index. **G** Dose-effective curve of the DFO + CHC treatment. **H** Mixture-algebraic estimate of the DFO + CHC treatment, CI > 1 indicates antagonism, CI = 1 indicates additive, CI < 1 indicates synergism. **I**. **J** Evaluation of protein expression upon treatment with varying concentrations of DFO + CHC, using WB analysis (day 3)

As it had been suggested that enhanced glycolysis in response to DFO treatment is a compensatory mechanism operating in cells to improve the survival rate under conditions of DFO challenge, we also evaluated the changes in lactate concentration in response to various concentrations of DFO. A significant increase in lactate levels was observed in the culture medium upon treatment with DFO (Fig. 4A). As DFO triggers a metabolic shift toward glycolysis and increased lactate production, we employed combinatorial treatment with DFO and α-cyano-4-hydroxy cinnamate (CHC), which suppresses extracellular excretion of lactate, and found that combinatorial treatment with DFO and CHC suppressed cell proliferation (Fig. 4B). Furthermore, experiments using varying concentrations of CHC and DFO revealed that the combination of CHC and DFO considerably increased the anticancer effect relative to the monotherapies (Fig. 4C–E). Furthermore, the combination index (CI) was less than 1.0, suggesting that DFO and CHC act in a synergistic manner (Fig. 4F–H). Intracellular lactate level was increased by treating with CHC (Fig. 4I). Western blotting revealed that the combined use of DFO and CHC resulted in synergistic increase in the levels of cleaved PARP and activated caspase-3 proteins, which are proteins associated with apoptosis (Fig. 4J).

### Efficacy of the combination therapy depends on the degree of increase in lactate concentration by DFO treatment

**Fig. 5:**
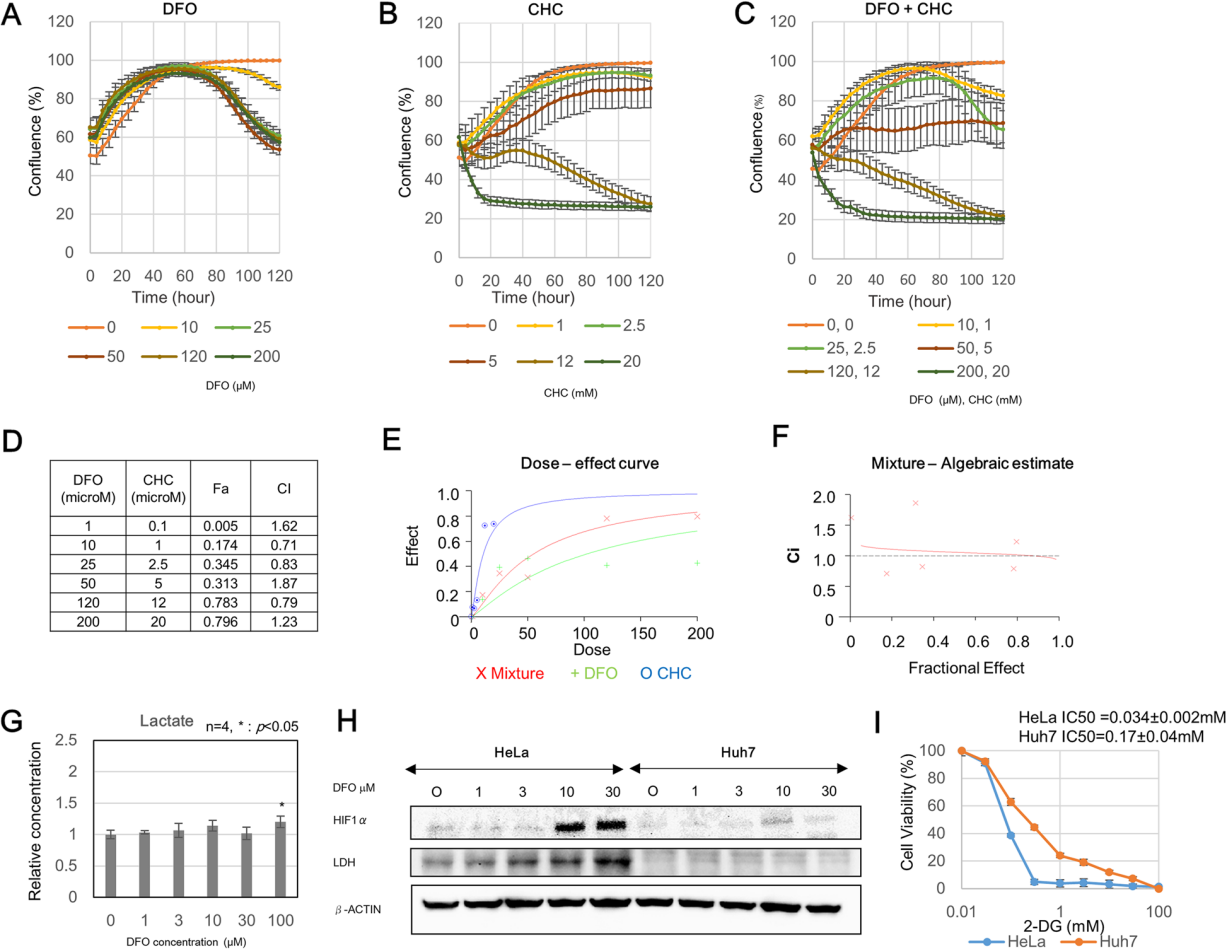
Evaluation of the effect of combined DFO + CHC treatment in Huh7 cells. **A** Changes in cell confluence observed after DFO monotherapy. **B** Changes in cell confluence observed after CHC monotherapy. **C** Changes in cell confluence observed after DFO + CHC treatment. **D** Combination index of DFO + CHC. Fa, fraction affected; CI, combination index. **E** Dose-effective curve of the DFO + CHC treatment. **F** Mixture-algebraic estimate of the DFO + CHC treatment. CI > 1 indicates antagonism, CI =1 indicates additive, CI < 1 indicates synergism. **G** Evaluation of lactate production upon varying DFO concentrations. Lactate concentration in the medium was measured by a lactose sensor (day 2). *Indicates *p* < 0.05 compared to control group (one-way ANOVA followed by Tukey’s post hoc test). **H** LDH expression in HeLa cells and Huh7 cells upon varying DFO concentrations (day3). **I** Cell viability assay of HeLa cells treated with 2-deoxyglucose (2-DG). IC50 values are 50% cell growth inhibitory concentrations of 2-DG on day 3. Cell viability was analyzed by MTS assay. IC50 was obtained from the following equation: IC50 = 10^[LOG(A/B) × (50-C)/(D-C) + LOG(B)]. **A** A higher concentration of two values that sandwich IC50. **B** A lower concentration of two values that sandwich IC50. **C** Cell viability (%) at (**B**). **D** Cell viability (%) at (**A**). Data are shown as mean ± SD

**Fig. 6:**
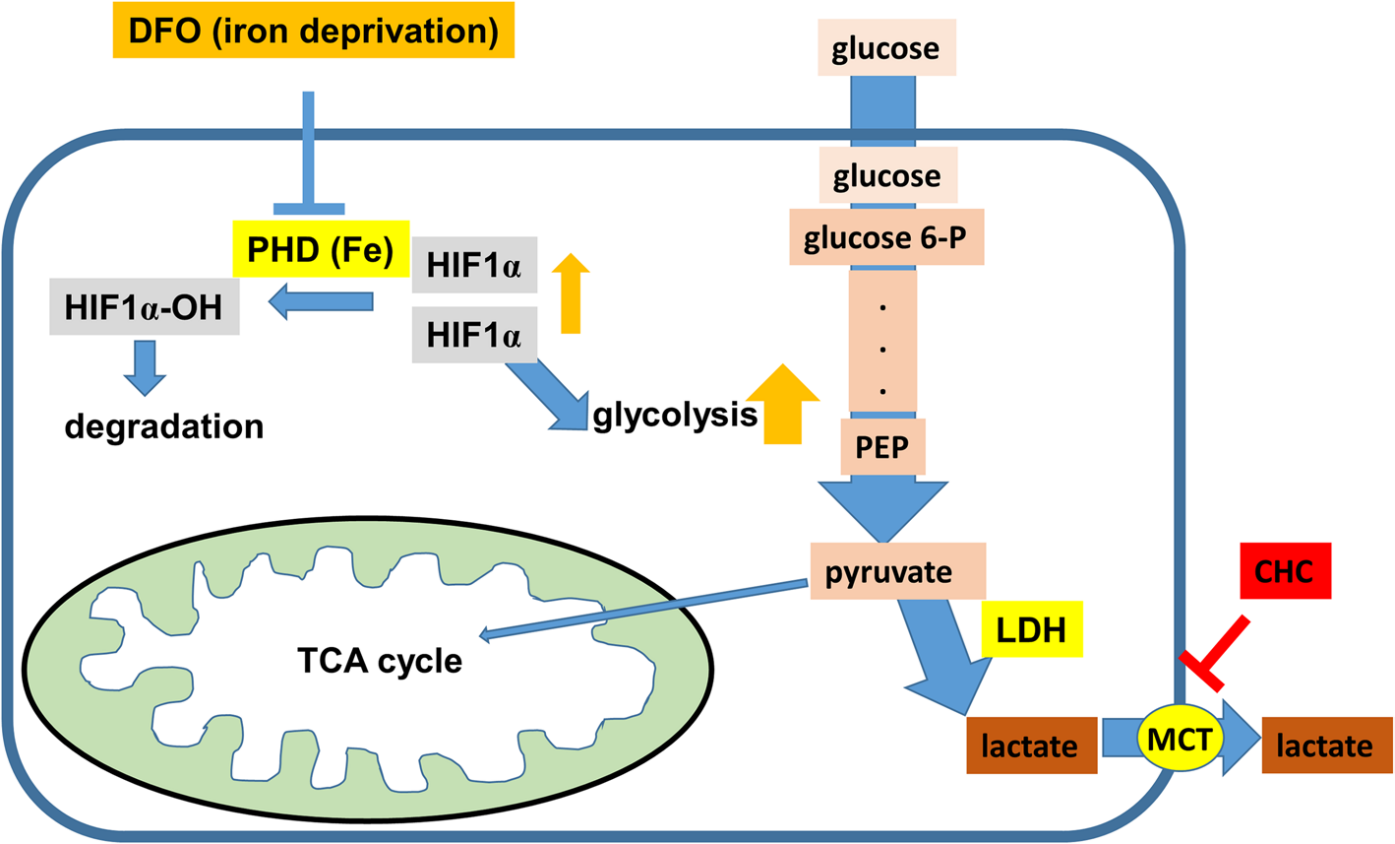
Schematic representation of the metabolic pathways altered by DFO administration and specific inhibitors. The iron chelator DFO is primarily taken up into cells by endocytosis. The chelation of iron inhibits PHD enzyme, leading to the accumulation of HIF1 α, thereby inducing a hypoxic response under normal oxygen conditions and increased production of lactate (final product of glycolysis). Overproduced lactate is excreted out of the cells by monocarboxylate transporter (MCT); however, suppression of this excretion process by CHC leads to accumulation of lactate in the cell. This increases the acidity inside the cell, which ultimately damages the cell

In order to examine whether an effect, similar to that observed in HeLa cells, is also achieved in liver cancer cell lines, we used various concentrations of DFO, CHC, and DFO + CHC for treating Huh7 cells and examined the combinatorial effect of the two agents (Fig. 5A–C). The combination indices (CI) were not below 1.0, which meant that combinatorial treatment with DFO and CHC did not have any effect on Huh7 cells (Fig. 5D–F). The inefficacy of the combinatorial treatment with DFO and CHC can be attributed to the relatively low accumulation of lactate in Huh7 cells than in HeLa cells, although lactate concentration did increase with increasing concentrations of DFO (Fig. 5G). Furthermore, Western blotting showed that HeLa cells exhibited marked accumulation of HIF1α and LDH with increasing DFO concentrations, whereas Huh7 cells exhibited reduced accumulation of HIF1α with no increase in LDH expression (Fig. 5H). We next evaluated the glycolysis dependency by assessing the IC50 of 2-DG and found that the IC50 of Huh7 was higher than that of HeLa, suggesting that the fact that Huh7 is less dependent on glycolysis than HeLa is one of the reasons for the lower expression of LDH and HIF1 in Huh7 (Fig. 5I). Figure 6 summarizes the metabolic changes induced by DFO treatment as well as the target molecules of the mentioned inhibitors.

## Discussion

In the present study, treatment of HeLa cells with DFO resulted in suppressed cell proliferation and reduced OCR. Gene expression analysis of cells revealed altered expression of genes involved in pathways related to mitochondrial dysfunction and cell cycle. It is known that enhanced glucose metabolism in the cytoplasm results in the rapid production of ATP—and other molecules—required for cell proliferation, and that this glycolytic shift is responsible for the suppression of apoptosis, cancer growth, and metastasis [17]. A switch from glycolytic to mitochondrial metabolism is thought to reduce the likelihood of cancer cell growth and infiltration. The drug dichloroacetate has been used to induce a shift in glucose metabolism from the cytoplasm (glycolysis) to the mitochondria (oxidative phosphorylation) [18, 19]. It is important to identify metabolic changes ensuing in cells in response to DFO treatment. Novel therapeutic targets may be identified based on these metabolic changes. In this study, we generated a hitherto unreported DFO-resistant cell line and analyzed the changes in global gene expression and metabolism occurring in cells exposed to DFO. However, it is reported that although fermentative glycolysis has long been considered as one of the major metabolic pathways that allows energy production and provides intermediates for the anabolic growth of cancer cells, it has become now evident that in contrast to prior beliefs, mitochondria play a key role in tumorigenesis [20]; further studies are needed.

Until date, studies on combinatorial anticancer therapy using iron chelators suggest that a combination of DFO and radiation therapy may be useful for breast cancer [21]. Desferal (deferoxamine) controls the expression of human copper transporter 1 and transferrin receptor 1 via Sp1, and it exhibited synergistic cytotoxicity in combination with oxaliplatin in human cervical cancer cells [22]. We have also previously reported the effect of a combinatorial therapy with an iron chelator and gemcitabine in pancreatic cancer cell lines and the potential of using DFO as a part of combination therapy [13]. As our experiment involving the treatment of HeLa cells with DFO indicated a shift toward a glycolysis-predominant metabolic profile, which is advantageous for cell survival, we used the pan-monocarboxylic acid transport inhibitor CHC to suppress lactate excretion. It has been reported that suppression of lactate excretion reduces the intracellular pH and induces cell death, thereby effectively injuring the side population and suppressing chemotaxis in multiple myeloma cells [23]. Aside from HeLa cells, the synergistic effect of CHC and DFO was observed in breast cancer cells (MCF7) as well (data not shown), but no synergistic effect was observed for these two agents in liver cancer Huh7 cells. This was due to the relatively low accumulation of HIF1α in Huh7 cells upon DFO treatment than that in HeLa cells. This result revealed that low amount of lactate was produced in Huh7 cells, and thus, the suppression of extracellular secretion of lactate did not yield a synergistic effect. Furthermore, we treated Huh7 cells with phenformin, which is known to cause enhanced lactate production as a side effect; however, it did not sufficiently increase the lactate levels (data not shown). Furthermore, it has been reported that cells with low IC50 to 2-DG have high glucose dependency, and cells with low glucose dependency take up glutamine and efficiently produce ATP in mitochondria [24]. We speculated that there was no significant increase in lactate production in Huh7 cells due to a reduced metabolic activity stemming from the suppressed cell proliferation and glucose dependency. Therefore, it is essential to pre-evaluate the extent of glycolytic enhancement—particularly with respect to lactate production and glycolysis dependency—to identify vulnerable cells in which combinatorial treatment with CHC and DFO will be effective.

When administered intravenously, DFO exhibits a short plasma elimination half-life of around 10 min, and it is subsequently excreted through urine and fecal matter. For this reason, studies on the sustained-release of DFO using biodegradable hydrogels are underway [25]. Uptake of DFO by cells proceeds via endocytosis after which the compound is primarily localized in the cytoplasm [26]. However, there are some iron chelators that localize in different cellular compartments, such as DFX, deferiprone, and Dp44mt, and reports indicate that combinatorial treatment with deferiprone and DFO results in a more effective removal of iron from cells [27]. We expect that an enhanced anticancer effect can be achieved by altering the method of DFO administration and using other types of iron chelators.

## Conclusion

Taken together, we established a DFO-resistant cell line and delineated the mechanisms underlying DFO resistance by using metabolome and gene expression analyses. The combination therapy of DFO and CHC is effective in HeLa cells where DFO treatment results in elevated lactate levels. Our results illustrated that the components of the glycolytic pathway may serve as specific targets for developing an efficient anticancer combinatorial therapy using DFO. These findings will be beneficial for the development of novel cancer chemotherapeutics.

## Supplementary Information

**Additional file 1: Supplemental Table 1.**

## Data Availability

Not applicable.

## Abbreviations

DFO: Deferoxamine
OCR: Oxygen consumption rate
CHC: α-Cyano-4-hydroxycinnamate
CQ: Chloroquine

## References

[1] Salminen A, Kauppinen A, Kaarniranta K (2015). 2-Oxoglutarate-dependent dioxygenases are sensors of energy metabolism, oxygen availability, and iron homeostasis: potential role in the regulation of aging process. Cell Mol Life Sci.

[2] Sakaida I, Kyle ME, Farber JL (1990). Autophagic degradation of protein generates a pool of ferric iron required for the killing of cultured hepatocytes by an oxidative stress. Mol Pharmacol.

[3] Yu Y, Wong J, Lovejoy DB, Kalinowski DS, Richardson DR (2006). Chelators at the cancer coalface: desferrioxamine to Triapine and beyond. Clin Cancer Res.

[4] Umemura M, Kim JH, Aoyama H, Hoshino Y, Fukumura H, Nakakaji R, Sato I, Ohtake M, Akimoto T, Narikawa M, et al. The iron chelating agent, deferoxamine detoxifies Fe(Salen)-induced cytotoxicity. J Pharmacol Sci. 2017;134(4):203-10. 10.1016/j.jphs.2017.07.002 28779994

[5] Lui GY, Kovacevic Z, Richardson V, Merlot AM, Kalinowski DS, Richardson DR (2015). Targeting cancer by binding iron: dissecting cellular signaling pathways. Oncotarget.

[6] Sakaida I, Hironaka K, Uchida K, Okita K (1999). Iron chelator deferoxamine reduces preneoplastic lesions in liver induced by choline-deficient L-amino acid-defined diet in rats. Dig Dis Sci.

[7] Saeki I, Yamamoto N, Yamasaki T, Takami T, Maeda M, Fujisawa K, Iwamoto T, Matsumoto T, Hidaka I, Ishikawa T, et al. Effects of an oral iron chelator, deferasirox, on advanced hepatocellular carcinoma. World J Gastroenterol. 2016;22(40):8967–77. 10.3748/wjg.v22.i40.8967 PMC5083802 27833388

[8] Yamasaki T, Terai S, Sakaida I (2011). Deferoxamine for advanced hepatocellular carcinoma. N Engl J Med.

[9] Harima H, Kaino S, Takami T, Shinoda S, Matsumoto T, Fujisawa K, Yamamoto N, Yamasaki T, Sakaida I. Deferasirox, a novel oral iron chelator, shows antiproliferative activity against pancreatic cancer in vitro and in vivo. BMC Cancer. 2016;16:702. 10.1186/s12885-016-2744-9 PMC5007806 27582255

[10] Yu R, Wang D, Ren X, Zeng L, Liu Y. The growth-inhibitory and apoptosis-inducing effect of deferoxamine combined with arsenic trioxide on HL-60 xenografts in nude mice. Leuk Res. 2014;38(9):1085–90. 10.1016/j.leukres.2014.05.005 24908354

[11] Piro E, Lentini M, Levato L, Russo A, Molica S (2018). Sustained erythroid response in a patient with myelofibrosis receiving concomitant treatment with ruxolitinib and deferasirox. Chemotherapy.

[12] Tury S, Assayag F, Bonin F, Chateau-Joubert S, Servely JL, Vacher S, Becette V, Caly M, Rapinat A, Gentien D, et al. The iron chelator deferasirox synergises with chemotherapy to treat triple-negative breast cancers. J Pathol. 2018;246(1):103–14. 10.1002/path.5104 29876931

[13] Shinoda S, Kaino S, Amano S, Harima H, Matsumoto T, Fujisawa K, Takami T, Yamamoto N, Yamasaki T, Sakaida I. Deferasirox, an oral iron chelator, with gemcitabine synergistically inhibits pancreatic cancer cell growth in vitro and in vivo. Oncotarget. 2018;9(47):28434–44. 10.18632/oncotarget.25421 PMC6033369 29983871

[14] Saeki I, Terai S, Fujisawa K, Takami T, Yamamoto N, Matsumoto T, Hirose Y, Murata Y, Yamasaki T, Sakaida I. Bortezomib induces tumor-specific cell death and growth inhibition in hepatocellular carcinoma and improves liver fibrosis. J Gastroenterol. 2013;48(6):738–50. 10.1007/s00535-012-0675-z 23011081

[15] Shin SY, Fauman EB, Petersen AK, Krumsiek J, Santos R, Huang J, Arnold M, Erte I, Forgetta V, Yang TP, et al. An atlas of genetic influences on human blood metabolites. Nat Genet. 2014;46(6):543–50. 10.1038/ng.2982 PMC4064254 24816252

[16] Velculescu VE, Zhang L, Vogelstein B, Kinzler KW (1995). Serial analysis of gene expression. Science.

[17] Spencer NY, Stanton RC (2019). The Warburg effect, lactate, and nearly a century of trying to cure cancer. Semin Nephrol.

[18] Michelakis ED, Webster L, Mackey JR (2008). Dichloroacetate (DCA) as a potential metabolic-targeting therapy for cancer. Br J Cancer.

[19] Stacpoole PW. Therapeutic targeting of the pyruvate dehydrogenase complex/pyruvate dehydrogenase kinase (PDC/PDK) axis in cancer. J Natl Cancer Inst. 2017;109(11):1-14. 10.1093/jnci/djx071 29059435

[20] Cassim S, Vucetic M, Zdralevic M, Pouyssegur J. Warburg and Beyond: The Power of Mitochondrial Metabolism to Collaborate or Replace Fermentative Glycolysis in Cancer. Cancers. 2020;12(5):1119-41. 10.3390/cancers12051119 PMC7281550 32365833

[21] Lynn JV, Urlaub KM, Ranganathan K, Donneys A, Nelson NS, Subramanian C, Cohen MS, Buchman SR. The role of deferoxamine in irradiated breast reconstruction: a study of oncologic safety. Plast Reconstr Surg. 2019;143(6):1666–76. 10.1097/PRS.0000000000005647 PMC6538447 30907808

[22] Chen SJ, Kuo CC, Pan HY, Tsou TC, Yeh SC, Chang JY (2016). Desferal regulates hCtr1 and transferrin receptor expression through Sp1 and exhibits synergistic cytotoxicity with platinum drugs in oxaliplatin-resistant human cervical cancer cells in vitro and in vivo. Oncotarget.

[23] Hanson DJ, Nakamura S, Amachi R, Hiasa M, Oda A, Tsuji D, Itoh K, Harada T, Horikawa K, Teramachi J, et al. Effective impairment of myeloma cells and their progenitors by blockade of monocarboxylate transportation. Oncotarget. 2015;6(32):33568–86. 10.18632/oncotarget.5598 PMC4741786 26384349

[24] Pusapati RV, Daemen A, Wilson C, Sandoval W, Gao M, Haley B, Baudy AR, Hatzivassiliou G, Evangelista M, Settleman J. mTORC1-Dependent Metabolic Reprogramming Underlies Escape from Glycolysis Addiction in Cancer Cells. Cancer cell. 2016;29(4):548-62. 10.1016/j.ccell.2016.02.018 27052953

[25] Saito T, Tabata Y. Hypoxia-induced angiogenesis is increased by the controlled release of deferoxiamine from gelatin hydrogels. Acta biomaterialia. 2014;10(8):3641-9. 10.1016/j.actbio.2014.04.021 24769115

[26] Doulias PT, Christoforidis S, Brunk UT, Galaris D (2003). Endosomal and lysosomal effects of desferrioxamine: protection of HeLa cells from hydrogen peroxide-induced DNA damage and induction of cell-cycle arrest. Free Radic Biol Med.

[27] Hoffbrand AV, Taher A, Cappellini MD (2012). How I treat transfusional iron overload. Blood.

