# Supplementary material for: An iron chelation-based combinatorial anticancer therapy comprising deferoxamine and a lactate excretion inhibitor inhibits the proliferation of cancer cells
Source: Cancer Metab. 2022 May 12;10:8. doi: 10.1186/s40170-022-00284-x (PMC9103045; doi:10.1186/s40170-022-00284-x)
Supplement: Supplementary file 1 — Additional file 1: Supplemental Table 1. [file 40170_2022_284_MOESM1_ESM.docx]

**Supplemental TABLE 1**

| Antibody | SOURCE | Catalogue number |
| --- | --- | --- |
| Rabbit monoclonal anti-PARP | Cell Signaling Technology | #5625S |
| Rabbit monoclonal anti-CASP3 | Cell Signaling Technology | #9661S |
| Rabbit monoclonal anti-HIF1α | Cell Signaling Technology | #3716S |
| Mouse monoclonal anti-βACTIN | SIGMA | 051M4892 |
| Anti rabbit IgG, Horseradish peroxidase linked whole antibody | cytiva | NA934V |
| Anti mouse IgG, Horseradish peroxidase linked whole antibody | cytiva | NA9310V |
| Chemicals | SOURCE | Catalogue number |
| CHC | Sigma-Aldrich, Tokyo, Japan | C2020 |
| DFO | Novartis Pharma K.K., Tokyo, Japan | D01186 |
